# Human gut bifidobacteria inhibit the growth of the opportunistic fungal pathogen *Candida albicans*

**DOI:** 10.1093/femsec/fiac095

**Published:** 2022-08-25

**Authors:** Liviana Ricci, Joanna Mackie, Gillian E Donachie, Ambre Chapuis, Kristýna Mezerová, Megan D Lenardon, Alistair J P Brown, Sylvia H Duncan, Alan W Walker

**Affiliations:** Rowett Institute, University of Aberdeen, Aberdeen, AB25 2ZD, United Kingdom; CIBIO - Department of Cellular, Computational and Integrative Biology, University of Trento, Trento, 38123, Italy; Aberdeen Fungal Group, Institute of Medical Sciences, University of Aberdeen, Aberdeen, AB25 2ZD, United Kingdom; Rowett Institute, University of Aberdeen, Aberdeen, AB25 2ZD, United Kingdom; Aberdeen Fungal Group, Institute of Medical Sciences, University of Aberdeen, Aberdeen, AB25 2ZD, United Kingdom; Department of Microbiology, Faculty of Medicine and Dentistry, Palacký University Olomouc, Olomouc, 77515, Czech Republic; Aberdeen Fungal Group, Institute of Medical Sciences, University of Aberdeen, Aberdeen, AB25 2ZD, United Kingdom; School of Biotechnology and Biomolecular Sciences, University of New South Wales, Sydney, NSW 2052, Australia; Aberdeen Fungal Group, Institute of Medical Sciences, University of Aberdeen, Aberdeen, AB25 2ZD, United Kingdom; MRC Centre for Medical Mycology, University of Exeter, Exeter, EX4 4QD, United Kingdom; Rowett Institute, University of Aberdeen, Aberdeen, AB25 2ZD, United Kingdom; Rowett Institute, University of Aberdeen, Aberdeen, AB25 2ZD, United Kingdom

**Keywords:** human gut microbiota, bifidobacteria, colonization resistance, *Candida albicans*, short chain fatty acids, lactate, pH

## Abstract

The human gut microbiota protects the host from invading pathogens and the overgrowth of indigenous opportunistic species via a process called colonization resistance. Here, we investigated the antagonistic activity of human gut bacteria towards *Candida albicans*, an opportunistic fungal pathogen that can cause severe infections in susceptible individuals. Coculture batch incubations of *C. albicans* in the presence of faecal microbiota from six healthy individuals revealed varying levels of inhibitory activity against *C. albicans*. 16S rRNA gene amplicon profiling of these faecal coculture bacterial communities showed that the *Bifidobacteriaceae* family, and *Bifidobacterium adolescentis* in particular, were most correlated with antagonistic activity against *C. albicans*. Follow-up mechanistic studies performed under anaerobic conditions confirmed that culture supernatants of *Bifidobacterium* species, particularly *B. adolescentis*, inhibited *C. albicans in vitro*. Fermentation acids (FA), including acetate and lactate, present in the bifidobacterial supernatants were important contributors to inhibitory activity. However, increasing the pH of both bacterial supernatants and mixtures of FA reduced their anti-*Candida* effects, indicating a combinatorial effect of prevailing pH and FA. This work, therefore, demonstrates potential mechanisms underpinning gut microbiome-mediated colonization resistance against *C. albicans*, and identifies particularly inhibitory components such as bifidobacteria and FA as targets for further study.

## Teaser

This study showed that faecal microbiota vary in their ability to inhibit the fungal pathogen *Candida albicans*, and identified bifidobacteria, and their fermentation acids, as inhibitory gut microbiota components.

## Introduction

The human colon harbours a diverse microbiota that is dominated by obligate anaerobic bacteria (Pasolli et al. 2019, Whitman et al. 1998). The main energy sources for these gut microbes are nondigestible carbohydrates that resist digestion in the small intestine and become available for bacterial fermentation in the proximal colon (Flint et al. 2015). These substrates are fermented by the gut microbiota to produce short-chain fatty acids (SCFAs), such as acetate, propionate, and butyrate, and other fermentation acids (FA) such as lactate (Cummings 1981). SCFAs provide the host with up to 5%–10% of their total daily energy requirement (Mortensen and Clausen 1996), and positively impact intestinal and systemic host health (Cummings 1981, Koh et al*.*^2016^). ‬‬‬‬‬‬‬‬‬‬‬‬‬‬‬‬‬‬‬‬‬‬‬‬‬‬‬‬‬‬‬‬‬‬‬‬‬‬‬‬‬‬‬‬‬‬‬‬

The intestinal microbiota also contributes to host health by bolstering resistance against colonization of the gut by pathogens (Bohnhoff et al. 1964, Buffie et al. 2015). This phenomenon, termed colonization resistance, can prevent pathogens from establishing and replicating in the gut, or from reaching the densities required to invade deeper tissues and cause overt disease (Bohnhoff et al. 1964). Colonization resistance is multifactorial, involving mechanisms such as the direct production of antimicrobial compounds (Donia and Fischbach 2015, Rea et al. 2010), competition for adhesion receptors on the gut epithelium (Ventura et al. 2016), and direct competition for niches and nutrients required for the growth of competing pathogenic bacteria (Deriu et al. 2013, Freter et al. 1983, Maltby et al. 2013, Wilson and Perini 1988). Additional mechanisms of colonization resistance include the creation of a less favourable gut environment, e.g. by lowering the luminal pH through the production of SCFAs (Cherrington et al. 1991, Rivera-Chávez et al. 2016, Roe et al. 2002), or depleting free molecular oxygen, which can prevent overgrowth and virulence gene expression of some pathogenic microbes (Marteyn et al. 2011, Rivera-Chávez et al. 2016). Furthermore, human gut commensals are instrumental in the training and modulation of the host immune system (Kau et al. 2011, Thaiss et al. 2016), inducing the release of host antimicrobial compounds (Cash et al. 2006, Fan et al. 2015), and in stimulating epithelial barrier reinforcement and repair (Geirnaert et al. 2017, Rossi et al. 2015). Importantly, microbiota-mediated colonization resistance can be weakened by various environmental factors and insults, such as Western-style diet (Martinez-Medina et al. 2014), antibiotic therapy (Bohnhoff et al. 1964, Vollaard et al. 1992), and acute and chronic inflammatory conditions (Carroll et al. 2012, Stecher et al. 2007).

*Candida albicans* is a diploid polymorphic fungus and a common opportunistic pathogen of humans, with an estimated annual incidence of 700 000 cases of *Candida* bloodstream infections globally (Guinea 2014). In susceptible patient cohorts, including premature infants and those undergoing chemo- or immune-therapy, organ or stem cell transplants, or abdominal surgery or trauma, *C. albicans* infections can be particularly devastating, with mortality rates of 46%–75% following systemic spread, even with antifungal drug interventions (Brown et al. 2012). The incidence of *C. albicans* infections has increased in vulnerable subjects over the past few decades (Low and Rotstein 2011) alongside the emergence of other clinically important *Candida* spp., such as *C. auris* (Heaney et al. 2020, Pfaller et al. 2000). Furthermore, a significant increase of isolates with resistance to common antifungal agents has been observed (Whaley et al. 2016).

Despite the pathogenic potential of *C. albicans*, it exists harmlessly in the gastrointestinal tract (GIT) of 40%–80% of healthy individuals in Western countries, predominantly in the yeast form, and with cell counts that do not typically exceed 10^4–5^ colony forming units (CFU)/g faeces (Harnett et al. 2017, Mason et al. 2012, Nash et al. 2017, Neville et al. 2015, Odds et al. 1989). The GIT is therefore a natural reservoir of *C. albicans* (Hube 2004, Odds 2010) but, in health, its overgrowth is suppressed by the gut microbiota via colonization resistance (Fan et al. 2015, Kennedy and Volz 1985a). However, conditions such as weakened immunity, increased permeability of the intestinal mucosal barrier, and/or perturbation of microbiota-mediated colonization resistance via receipt of broad-spectrum antibiotics can favour *C. albicans* pathogenesis (d'Enfert et al. 2020, León et al. 2009, Samonis et al. 1994,). Furthermore, systemic candidiasis is often reported to derive from a preceding expansion of *Candida* spp. in the GIT and subsequent translocation from the intestinal niche into the bloodstream (Miranda et al. 2009, Zhai et al. 2020). GIT colonization by *C. albicans* is, therefore, a major risk factor for systemic candidiasis (Pittet et al. 1994).

Given the importance of the intestinal niche as a reservoir for systemic dissemination, and the known suppressive effects of the indigenous microbiota on the colonization of the gut by *C. albicans* in health (Fan et al. 2015), we here assessed the potential of the human gut microbiota, and individual gut anaerobe species, to suppress the growth of this opportunistic pathogen *in vitro*. We identified specific bacterial isolates, including *Bifidobacterium adolescentis*, in faecal samples of healthy individuals that inhibit *C. albicans* growth *in vitro*, and revealed the involvement of gut bacterial FA and pH in this process. These findings enhance current knowledge on potential mechanisms of colonization resistance against *C. albicans* in the human gut, and suggest targets for further studies that aim to utilize the gut microbiota as a source of novel therapies with antagonistic activity against this opportunistic fungal pathogen.

## Materials and methods

### Ethics

Faecal sample collections used for isolation of human gut anaerobes, and for coculture experiments with *C. albicans*, were approved by the Ethical Review Panel of the Rowett Institute under study number 5946. No donors had received antibiotic treatment for at least 6 months prior to faecal donation.

### Cultivation of *C. albicans* strain SC5314

*Candida albicans* strain SC5314 (Gillum et al. 1984) was prepared by plating 2–10 µl of frozen glycerol stock on YPD plates [1% w/v yeast extract (Oxoid LP0021, Basingstoke, UK), 2% w/v mycological peptone (Oxoid LP0040), 2% w/v d-glucose, and 2% w/v agar No. 2 (Oxoid LP0012)] and incubating at 30°C for 48 h. A single colony was transferred from the Petri dish into NGY broth (0.1% yeast extract (Oxoid LP0021), 0.1% neopeptone (Difco, Franklin Lakes, NJ, USA), and 0.4% w/v d-glucose; MacCallum et al. 2006) and incubated at 30°C, with shaking at 200 rpm, overnight. The concentration of *C. albicans* cells in suspension (cells/ml) was estimated by counting using a haemocytometer. Yeast growth was assessed by measuring optical density of the cultures at a wavelength of 600 nm using a spectrophotometer. For determination of *C. albicans* CFUs in samples, cells were plated on Sabouraud dextrose agar (SDA; 4% w/v d-glucose, 1% w/v mycological peptone, and 2% w/v agar No. 2, pH 5.6).

### Batch cocultures of *C. albicans* and mixed faecal microbiota from healthy donors

Cocultures of *C. albicans* and mixed faecal microbiota were performed in duplicate for each faecal donor in anaerobically sealed Wheaton bottles containing complex anaerobic medium. The medium contained (amounts given are for 1 l): oat spelt xylan (0.6 g; Sigma-Aldrich, St. Louis, MO, USA), pectin (citrus, 0.6 g; Sigma-Aldrich), amylopectin (0.6 g; Sigma-Aldrich), arabinogalactan (larch, 0.6 g; Sigma-Aldrich), potato starch (5.0 g; Sigma-Aldrich), inulin (0.6 g; Sigma-Aldrich), porcine mucin (0.5 g; Sigma-Aldrich), casein hydrolysate (0.5 g; Fluka, Charlotte, NC, USA), peptone water (0.5 g; Oxoid), K_2_HPO_4_ (2.0 g; BDH, Dubai, UAE), NaHCO_3_ (0.2 g; Sigma-Aldrich), NaCl (4.5 g; Fisher Scientific), MgSO_4_ · 7H_2_O (0.5 g; BDH), CaCl_2_ · 2H_2_O (0.45 g; Sigma-Aldrich), FeSO_4_ · 7H_2_O (0.005 g; Hopkin & Williams, UK), haemin (0.01 g; Sigma-Aldrich), bile salts (0.05 g, Oxoid), 0.1% w/v resazurin (0.6 ml), antifoam A (Y-30, 0.5 ml; Sigma-Aldrich), and dH_2_O to 1 l. In addition, the medium was supplemented with filter-sterilized reducing solution to ensure anaerobic conditions (0.5 g cysteine, 3.0 g NaHCO_3_, and dH_2_O to 40 ml). The pH was adjusted to 6.5 (using HCl and NaOH, as appropriate) before dispensing the medium (50 ml aliquots) into Wheaton bottles anaerobically and autoclaving. After autoclaving, Wheaton bottles were supplemented with 100 µl mineral solution (150 mg EDTA, 60 mg FeSO_4_ · 7H_2_O, 3.0 mg ZnSO_4_ · 7H_2_O, 0.9 mg MnCl_2_ · 7H_2_O, 9.0 mg boric acid, 6.0 mg CoCl_2_ · 6H_2_O, 0.3 mg CuCl_2_ · 2H_2_O, 0.6 mg NiCl_2_ · 6H_2_O, 0.9 mg NaMoO_4_ · 2H_2_O, and dH_2_O to 300 ml), 70 µl vitamin solution (0.2 g menadione, 0.4 g biotin, 0.4 g pantothenate, 2.0 g nicotinamide, 0.1 g vitamin B_12_, 0.8 g thiamine, 1.0 g *p*-aminobenzoic acid, and dH_2_O to 200 ml), 155 µl of a SCFA solution (17 ml acetic acid, 6ml propionic acid, 1 ml n-valeric acid, 1 ml *iso*-valeric acid, 1 ml *iso*-butyric acid, and 5 ml butyric acid) and 153 µl of a solution containing additional medium components (2 μg folic acid, 2000 μg inositol, 400 μg niacin, 400 μg pyridoxine HCl, 200 μg riboflavin, 100 μg potassium iodide, and 200 μg ferric chloride and dH_2_O to 1 l).

*Candida albicans* cells from an overnight culture grown in YPD broth were washed in sterile PBS, counted using a haemocytometer, and inoculated into 50 ml anaerobic media in Wheaton bottles at a final concentration of 5 × 10^6^ cells/ml (except for one pilot experiment where the inoculum was 5 × 10^5^ cells/ml, see ‘Results’ section for more details). Faecal samples were obtained from six different donors and slurries (10% w/v faeces) were prepared in gentleMACS™ M tubes (Miltenyi Biotech, Auburn, CA, USA) by homogenization in anaerobic PBS (PBS containing 0.05% cysteine). Faecal homogenates were centrifuged at 500 x *g* for 5 min and the liquid faecal component was injected into the Wheaton bottles using a sterile syringe to give a 0.02% faecal suspension at baseline. The inoculated Wheaton bottles were incubated at 35°C for 48 h with gentle shaking at 75 rpm. Measurements of *C. albicans* CFUs were carried out at *t* = 0, 24, and 48 h by plating 10-fold serial dilutions on SDA plates supplemented with 34 µg/ml chloramphenicol. CFUs were counted after aerobic incubation at 30°C for 2–3 days.

### 16S rRNA gene amplicon sequencing of cocultured incubation samples

The faecal inocula from healthy donors used in the coculture experiments, and from the two biological replicate samples collected after 24 and 48 h of incubation with *C. albicans*, were analyzed by Illumina MiSeq-based 16S rRNA gene profiling, targeting the V1–V2 region of the gene. Genomic DNA was extracted using the FastDNA^TM^ SPIN Kit for Soil (MP Biomedicals, Irvine, CA, USA) following the manufacturer's instructions. Barcoded fusion primers containing adaptors for downstream Illumina MiSeq sequencing MiSeq-27F (5′-AATGATACGGCGACCACCGAGATCTACACTATGGTAATTCCAGMGTTYGATYMTGGCTCAG-3′) and MiSeq-338R (5′-CAAGCAGAAGACGGCATACGAGAT-barcode-AGTCAGTCAGAAGCTGCCTCCCGTAGGAGT-3′) were used for PCR amplification of 16S rRNA genes from extracted DNA. PCR was performed using Q5 Taq polymerase (New England Biolabs, Ipswich, MA), with the following cycling conditions: 98°C for 2 min; followed by 20 cycles at 98°C for 30 s, 50°C for 30 s, and 72°C for 90 s; with a final extension at 72°C for 5 min. Each sample was amplified in quadruplicate; the four reactions were pooled, and PCR products were ethanol precipitated to generate a single PCR amplicon tube per sample. These PCR products were then quantified using a Qubit 2.0 fluorometer (Life Technologies, Carlsbad, CA, USA), and a sequencing master mix was prepared by mixing the samples in equimolar amounts, which was then sequenced at the Centre for Genome-Enabled Biology and Medicine (CGEBM) at the University of Aberdeen (Aberdeen, UK). For sequencing, an Illumina MiSeq machine was used, with 2 × 250 bp read length. The raw output sequence data are available from the European Nucleotide Archive, under the project accession number PRJEB48351. Individual sample accession numbers are given in Table S1 (Supporting Information).

### Analysis of 16S rRNA gene amplicon data

The raw read data in fastq format were analyzed using the open-source software Mothur (Schloss et al. 2009). For both of the timepoints after coculture, the two experimental replicates were pooled into single samples for final analyses as no statistically significant differences were detected between replicates. Briefly, contigs were created using the make.contigs command and low quality contigs (such as with length < 280 or > 470 bases, containing at least one ‘N’, and polymeric stretches > 7 bases) were filtered out using screen.seqs. The contigs were aligned against the SILVA reference (https://www.arb-silva.de/; Quast et al. 2013), and operational taxonomic units (OTUs) were generated at a 97% similarity cut-off level, with a preclustering step of diffs = 3 to reduce the impact of sequencing errors. Chimera removal software was not used as abundant OTUs corresponding to bifidobacteria were mistaken for chimeric sequences. Instead, the split.abund command was used to filter out low-abundance sequences that appeared less than 10 times in the dataset. All samples were rarefied to 9171 reads for subsequent comparative analyses. Samples derived from the D1 and D3 faecal inocula samples generated far fewer reads than this, so were excluded from the final analyses. Taxonomic classifications were assigned to each OTU by mapping against the RDP reference database (Cole et al. 2014). Taxonomies for selected OTUs were also validated by manually checking representative sequences using BLAST searches against the NCBI nucleotide database (https://blast.ncbi.nlm.nih.gov/Blast.cgi), and the Ribosomal Database Project (Cole et al. 2014, Johnson et al. 2008). Alpha-diversity measures, and phylotype analyses at the phylum, family and genus levels were carried out using Mothur. The final OTU table, phylum, family, genus, and alpha-diversity results for each sample are shown in Table S1 (Supporting Information). The faecal and enriched microbial community coculture samples were assigned to the categories ‘benign’ or ‘antagonistic’ according to the extent of the inhibition shown against *C. albicans*. Putative biomarkers at different taxonomic levels that correlated with antagonistic activity against *C. albicans* were assessed using LEfSe (Segata et al. 2011), as implemented in Mothur.

### Culturing of human gut anaerobes

The gut anaerobes tested in the current study included isolates from the Rowett Institute (Aberdeen, UK) strain collection or purchased from DSMZ (Braunschweig, Germany) (Table S2, Supporting Information). The isolates were revived from stocks, anaerobically, in Hungate tubes containing M2GSC medium supplemented with 10% v/v clarified bovine rumen fluid (Bryant 1972, Miyazaki et al. 1997). Inoculated cultures were incubated at 37°C in a static 5% CO_2_ incubator overnight (NuAire, Plymouth, MN, USA). Cell growth was monitored by measuring optical density at 650 nm (OD_650_) using a spectrophotometer (Novaspec II, Amersham BioSciences UK Ltd., Little Chalfont, UK).

Some of the anaerobic bacteria tested for anti-*Candida* activity in this study were newly isolated from the stool samples of two consenting adults (D3 and DM1). For each donor, 10-fold serial faecal dilutions were prepared in M2 medium (Hobson 1969) with no added carbon source. Each preparation was then used to inoculate five different agar plates: fastidious anaerobe agar (FAA, LAB M Ltd, Heywood, UK) supplemented with 5% v/v horse blood and 0.5% w/v menadione; FAA supplemented with 5% v/v horse blood; brain heart infusion (BHI, Oxoid); M2GSC (Miyazaki et al. 1997); and M2GSC supplemented with 0.5% w/v haemin and 0.5% w/v menadione. The plates were incubated in an anaerobic cabinet (Don Whitley Scientific, Bingley, UK) for 48 h. In parallel, faecal dilutions were preincubated in M2-AXOS diluting broth [M2 supplemented with 0.2% w/v arabinoxylan oligosaccharides (Cargill, Wayzata, MN, USA)] before streaking. After 4 d of incubation, single colonies were selected and picked onto duplicate agar plates of the same type of culture medium on which they were first grown. Half of these duplicate plates were left to grow in the anaerobic cabinet, while the remaining plates were incubated aerobically, at 37°C, for up to 48 h. At the end of the incubation, the growth on anaerobic plates was compared with that on the aerobic counterparts to screen for strictly anaerobic isolates. Single colonies were picked from plates that only showed anaerobic growth and then grown in Hungate tubes containing either M2GSC medium supplemented with 0.5% w/v haemin and 0.5% w/v menadione, fastidious anaerobe broth supplemented with 5% v/v horse blood, and 0.5% w/v menadione, or BHI broth. DNA was extracted from the collected cultures using the FastDNA^TM^ SPIN Kit for Soil (MP Biomedicals) and 16S rRNA genes were amplified using universal bacterial primers (7F- AGAGTTTGATYMTGGCTCAG and 1510R- ACGGYTACCTTGTTACGACTT; Satokari et al. 2001) and Sanger sequenced (Eurofins Genomics) for taxonomic identification using BLAST (Johnson et al. 2008), and the Ribosomal Database Project Classifier (Cole et al. 2014). Culturing conditions used to obtain each of the novel isolates are shown in Table S3 (Supporting Information).

### Inhibition of *C. albicans* growth by gut bacterial supernatants and gut bacterial FA

In order to assess the effect of individual gut bacterial isolates on the growth of *C. albicans* strain SC5314, anaerobes of interest (Table S2, Supporting Information) were cultured in tubes with anaerobic M2GSC medium at 37°C overnight. The individual culture supernatants were then collected after centrifugation at 658 × *g* for 10 min. The supernatants were filter-sterilized by passing through 0.2 μm syringe-driven filter units (Millex, Merck Millipore Ltd, Kenilworth, NJ, USA) to remove residual bacterial cells. *Candida albicans* cells pregrown in NGY to an OD_600_ of 0.8–0.95 were diluted 1 in 100 in fresh NGY medium and 100 μl was transferred to wells of 96-well microtitre plates (CoStar, Washington, WA, USA). The *C. albicans* suspensions were incubated with an equal amount of filter-sterilized bacterial culture supernatant, or fresh NGY medium as a control, to assess the fungal growth, with technical replicates. The 96-well plates were incubated anaerobically in a temperature-controlled plate reader at 37°C (Epoch 2 Microplate Spectrophotometer, BioTek, Swindon, UK). For each test and technical replicate, the growth of *C. albicans* was calculated by subtracting the OD_600_ value at time 0 from that measured after 24 h (T24–T0). The percentage growth of the fungus in fresh NGY medium in the absence of bacterial supernatant was set as 100% growth reference for each repeat run, and uninoculated filter-sterilized M2GSC medium was used as a control.

The impact of gut bacterial FA on *C. albicans* growth was assessed by monitoring fungal growth in the presence of a mixed solution of 45 mM sodium acetate (Sigma-Aldrich), 15 mM lactate (Sigma-Aldrich), and 10 mM sodium formate (VWR BDH Chemicals, Merck), supplemented with 0.4% w/v glucose, in addition to individual acids plus 0.4% w/v glucose. The pH of all solutions or NGY medium was adjusted using 1 M NaOH and 1 M HCl, as appropriate, to 4, 5, 6, or 7, and checked using a pH meter (Denver Instrument, Denver, CO, USA).

### Quantification of FA in gut bacterial culture supernatants using gas chromatography

The culture supernatants of the tested gut bacterial isolates were analyzed by capillary gas chromatography (GC) to quantify the production of FA. To determine the concentrations of SCFAs and lactate, the samples were first derivatized as described elsewhere (Richardson et al. 1989). Briefly, 1 ml of a culture supernatant was placed in a Sorvall screw-capped tube and 50 μl of 0.1 M 2-ethylbutyric acid was added as an internal standard. Concentrations of derivatized fatty acids were determined after a double step extraction of organic acids in 0.5 ml of HCl and 2 ml of diethyl ether per sample, and quantification of their tertiary butyldimethylsisyl (*t*-BDMS) derivatives using capillary GC apparatus (Agilent 6890; Agilent Technologies, Santa Clara). A total of two technical replicates of an external standard (acetic acid, propionic acid, *iso*-butyric acid, *n*-butyric acid, *iso*-valeric acid, *n*-valeric acid, sodium formate, lithium lactate, and sodium succinate) were analyzed alongside the samples in each GC run to assess quality of the extraction.

### Statistical analyses

The nonparametric Kruskal–Wallis test, followed by Dunn’s *post hoc* test, was used to analyze data from assays on the inhibition of *C. albicans* growth by gut bacterial supernatants, and to compare *C. albicans* growth in the absence and presence of gut anaerobe supernatants, using Prism v8.4.1 (GraphPad, San Diego, CA, USA). To test for associations between % *C. albicans* growth and the gut bacterial culture supernatants, a Spearman correlation was computed using Prism v8.4.1 (GraphPad). Exact *P*-values obtained using the Spearman correlation test were corrected using the two-stage linear step-up procedure of Benjamini, Krieger, and Yekutieli (false discovery rate approach, with Q = 5%). Parameters included the OD of microbial cultures, pH, and FA levels (acetate, formate, and lactate; separately and combined), as quantified in the culture supernatants using GC.

## Results

### Inhibitory activity of cultivated faecal microbiota on *C. albicans* growth varies between faecal donors

To establish whether the gut microbiota from different individuals vary in their ability to suppress the growth of *C. albicans*, we performed coculturing experiments in batch culture, where *C. albicans* SC5314 cells were incubated for up to 48 h alongside faecal inocula from six healthy adults. The cocultures were performed under anaerobic conditions in a complex growth medium designed to mimic the human colon environment. The viability of *C. albicans* cells was assessed by determining CFUs following plating onto SDA medium plus chloramphenicol at 0 h and after 24 h and 48 h incubations with or without homogenized faecal inocula.

**Figure 1.**
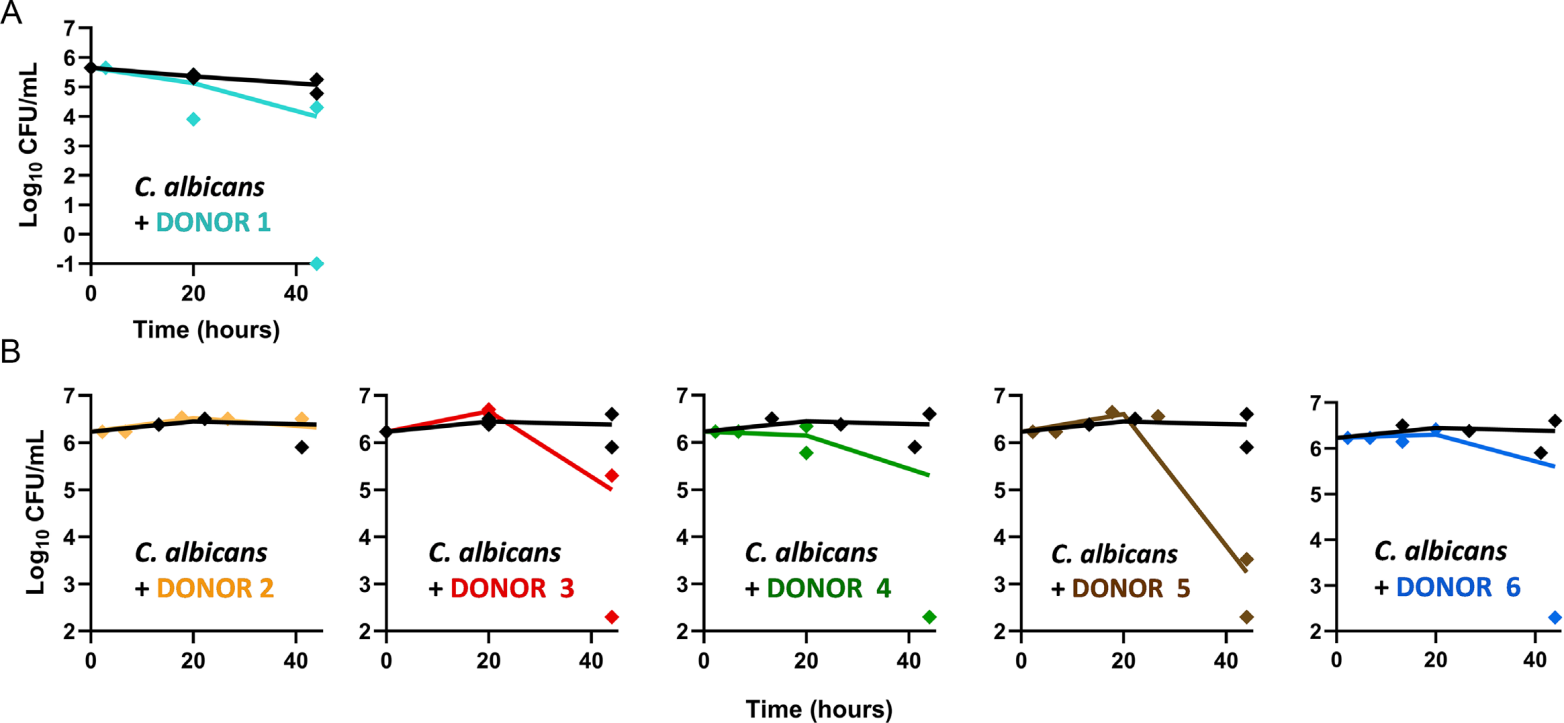
Faecal inocula from healthy donors resulted in varying killing activity against *C. albicans* cells *in vitro*. *Candida albicans* was cultured with faecal inocula from six different individuals (Donor 1–6), or with no faecal inocula as controls (black lines). Each data point (diamonds) represents *C. albicans* CFU/ml at sampled time points, while the line connects the means at each time point, calculated from two independent CFU measurements. Data were transformed to Log_10_ (*y*-axis). (A) *Candida albicans* was inoculated into the anaerobic medium at a density of 5 × 10^5^ cells/ml. (B) *Candida albicans* was inoculated into the anaerobic medium at a concentration of 5 × 10^6^ cells/ml.

An initial experiment was conducted with a stool sample from a single healthy volunteer (Donor 1). As shown in Fig. 1(A), the coculture of *C. albicans* (inoculated at 5 × 10^5^ cells/ml) with faecal material from Donor 1 showed a clear reduction in the fungal CFUs after 44 h incubation. However, viable cell counts were also reduced at the end of the control incubation when *C. albicans* was grown alone (Fig. 1, black lines), albeit the reduction was lower than that observed in coculture. Subsequent experiments, assessing the impact of faecal inocula from five additional donors were, therefore, performed using 10 times more *C. albicans* cells (inoculated at 5 × 10^6^ cells/ml), which was sufficient to maintain significant *C. albicans* CFUs throughout the experiments (Fig. 1B). In the control samples, without the faecal inoculum, *C. albicans* CFUs remained relatively constant throughout the 48 h incubation period, with counts around 2.5 × 10^6^ CFU/ml, indicating that the colon-mimicking growth medium and anaerobic conditions did not kill *C. albicans* (Fig. 1B, black lines). The experiment also revealed that the faecal microbiota from different individuals affected *C. albicans* viable counts to markedly differing degrees after 44 h of coculture (Fig. 1B, orange, red, green, brown, and blue lines). The faecal inoculum from Donor 5 resulted in the strongest inhibitory effect on *C. albicans* growth, with a 1000-fold (3-log) reduction of *Candida* CFUs at the end of the incubation period (1 × 10^3^ CFU/ml). Cocultures with faecal inocula from Donors 3, 4, and 6 also resulted in a decrease in *C. albicans* CFUs (between 4- and 20-fold decrease). In contrast, the faecal inoculum from Donor 2 resulted in no effect on *C. albicans* growth, which was comparable with that of the no faecal inoculum control, suggesting that the gut bacteria cultured from the faecal inoculum of this individual did not impair the fungal survival under the tested conditions. We conclude that the cultivated faecal samples from healthy individuals differed in their ability to inhibit the survival of *C. albicans*.

### Variance in faecal microbiota composition may impact colonization resistance against *C. albicans*

**Figure 2.**
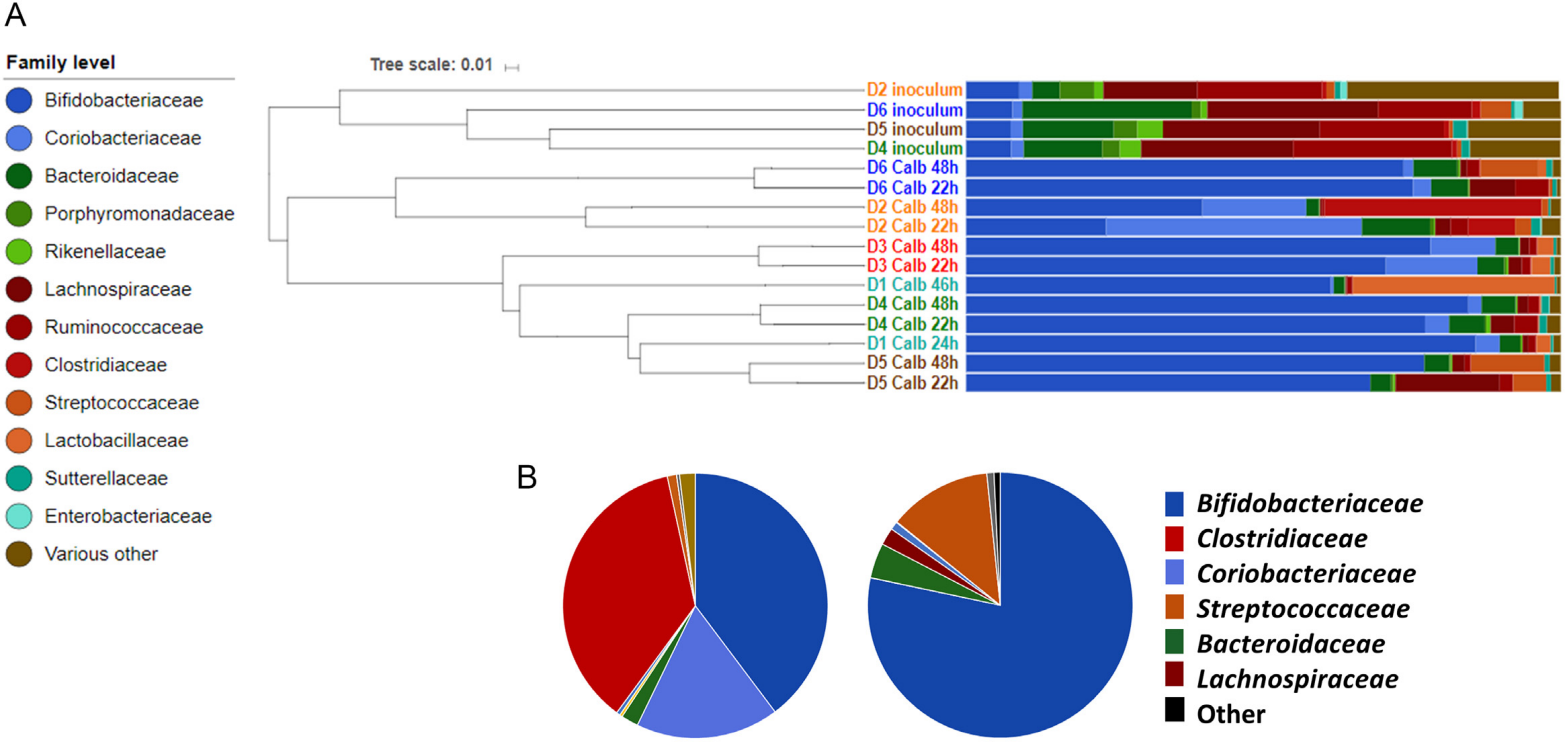
Bacterial community analysis of faecal samples and cocultures with varying inhibitory activity against *C. albicans*. (A) Bray–Curtis dendrogram of faecal inocula and subsequent cocultures with *C. albicans*. (B) Proportional family-level composition of Donor 2 (‘benign’) and Donor 5 (‘antagonistic’) faecal samples after 48 h coculture with *C. albicans* in anaerobic, colon-mimicking, medium.

The differing extent of *C. albicans* growth inhibition observed in cocultures with faecal inocula from different donors might result from differences in the cultured gut microbiota species composition and, consequently, their metabolic activities. Therefore, we used 16S rRNA gene amplicon-based sequence profiling to analyze the bacterial communities present in the initial faecal inocula from the different donors and in the coculture batch samples after 1 and 2 days of incubation. The analysis revealed that, as anticipated, at the OTU level, the initial faecal inoculum samples contained the highest alpha diversity, which then became reduced as certain bacterial taxa were selectively enriched during coincubation (Fig. 2A; Table S1, Supporting Information).

We classified the cultured faecal samples into different groups according to the observed impact on *C. albicans* growth in the batch coculture. Specifically, Donor 5 was defined as ‘antagonistic’ as the faecal inoculum from this donor resulted in the strongest inhibitory effect, as were Donors 1, 3, 4, and 6 (all > 85% *C. albicans* inhibition). The Donor 2 inoculum was classified as ‘benign’ since coincubation had comparatively little effect on *C. albicans* survival *in vitro* (12% inhibition).

The nonparametric analysis of molecular variance (AMOVA) test implemented in the Mothur software package (Schloss et al. 2009) was first used to compare the bacterial compositions of the cultivated ‘benign’ and ‘antagonistic’ samples (D2 v D1, 3, 4, 5, and 6) at days 1 and 2 combined, and revealed a statistically significant difference between the two groups (*P* = .02).

We next used LEfSe (Segata et al. 2011) to identify taxa that were associated with either the ‘antagonistic’ (D1, 3, 4, 5, and 6) or ‘benign’ status (D2). The analysis indicated that the *Bifidobacteriaceae* family (*P* = .032), and more specifically, *B. adolescentis* (*P* = .032) and *Bifidobacterium longum* derived OTUs (*P* = .032) belonging to the Gram-positive *Actinobacteria* phylum correlated with samples exerting the strongest antagonistic activity against *C. albicans* (Fig. 2B; Tables S4 and S5, Supporting Information). In contrast, the *Coriobacteriaceae* family (*P* = .032) and its constituent species *Collinsella aerofaciens* (*P* = .026; hereon, indicated as *Co. aerofaciens*), also belonging to the *Actinobacteria* phylum, together with *Clostridiaceae* (*P* = .031) and *Clostridium neonatale* (*P* = .026) from the *Firmicutes* phylum, correlated with the greatly reduced antagonistic activity against *C. albicans* (Fig. 2B; Tables S4 and S5, Supporting Information).

### Culture supernatants of specific human gut isolates inhibit *C. albicans* growth under anaerobic conditions

Having correlated the presence of bifidobacteria in the cultivated faecal samples with antagonistic activity against *C. albicans* using the 16S rRNA gene-based analysis, we next attempted to verify this finding by testing a panel of 37 common and dominant gut bacterial strains for inhibition of *C. albicans* growth *in vitro*. The original source of each bacterial isolate is shown in Table S2 (Supporting Information). A subset of the tested gut anaerobes was newly isolated for the purpose of this study from stool samples of healthy volunteers (see ‘Materials and Methods’ section for details of isolation steps), while other isolates were from the existing Rowett Institute strain collection or purchased from DSMZ. The species selected for these tests were also representative of the main phyla inhabiting the human gut (Table S2, Supporting Information). The bacterial isolates of interest therefore belonged to the phyla *Firmicutes* (nine strains belonging to the family *Lachnospiraceae*, four *Eubacteriaceae*, one *Peptostreptococcaceae*, three *Clostridiaceae*, six *Ruminococcaceae*, and one *Oscillospiraceae*), *Actinobacteria* (*B. adolescentis*, selected for analysis as this species was correlated with antagonist activity in coculture with *C. albicans*, and two *Coriobacteriaceae*), *Bacteroidetes* (five *Bacteroidaceae*, one *Porphyromonadaceae*, and two *Prevotellaceae*), and one *Proteobacteria* (*Enterobacteriaceae*).

**Figure 3.**
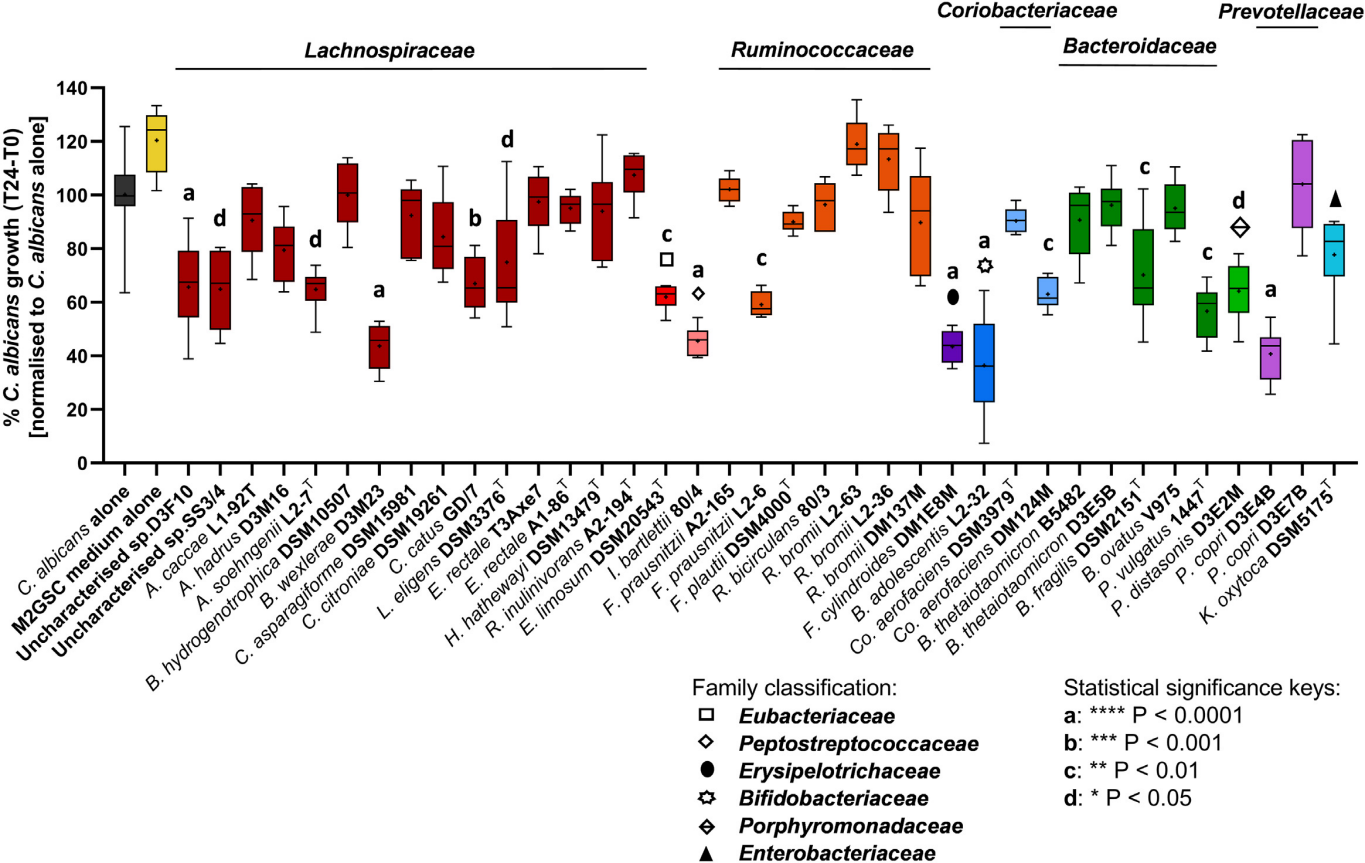
Impact of culture supernatants from individual human gut anaerobe strains on *C. albicans* growth under anaerobic conditions. The whisker boxplot represents % *C. albicans* growth (T24–T0) when incubated with pure culture supernatants from human gut isolates. The growth of *C. albicans* alone in fresh NGY medium (black) was monitored via six technical replicates per test (total *n* = 54). Strains are grouped by family and colour-coded: dark red for *Lachnospiraceae*; red for *Eubacteriaceae*; orange for *Ruminococcaceae*; purple for *Erysipelotrichia*; blue for *Bifidobacteriaceae*; light blue for *Coriobacteriaceae*; green for *Bacteroidaceae*; light green for *Porphyromonadaceae*; lilac for *Prevotellaceae*; and turquoise for *Enterobacteriaceae*. The cross represents the mean, while the central horizontal line shows the median of six technical replicates per strain (except for ‘Uncharacterized’ sp. D3F10, *n* = 17; *Coprococcus catus* GD/7 and *Lachnospira eligens* DSM 3376^T^, *n* = 12; *R. bromii* DM137M, *n* = 11; *B. adolescentis* L2-32, *n* = 24; and*Bacteroides fragilis* DSM 2151^T^, *n* = 11). The Kruskal–Wallis test revealed a highly significant difference between the effects of different supernatants (*P*< .0001), and Dunn’s *post hoc* identified multiple gut anaerobes whose culture supernatants significantly inhibited *C. albicans* growth compared to the *C. albicans*-only control, as indicated in the figure.

We reasoned that the inhibitory effects of gut microbes upon *C. albicans* might be mediated, at least in part, by secreted factors or metabolites. Therefore, in order to assess the putative *in vitro* inhibitory activity of the selected gut bacterial isolates, each species (Fig. 3) was grown individually in M2GSC liquid medium overnight. Then, filter-sterilized culture supernatant was incubated with an overnight liquid culture of *C. albicans* under anaerobic conditions for 24 h. *Candida albicans* biomass was assessed using optical density (OD_600_) measurements. The percentage growth of the fungus alone in fresh NGY medium, without exposure to bacterial supernatants, was set as 100% reference for each repeat run, and uninoculated M2GSC medium was used as a control.

The experiments revealed that the different supernatants varied widely in their effect on *C. albicans* growth (Fig. 3). Most of the isolates tested, including *Co. aerofaciens* DSM 3979^T^, which was correlated with ‘benign’ status in the earlier sequence-based profiling analysis, did not inhibit *C. albicans* growth. Of note, however, *Co. aerofaciens* strain DM124M showed a mild inhibitory effect (*P*< .01; Fig. 3), suggesting that the activity observed may be strain specific. In contrast, the *Blautia wexlerae* D3M23, *Faecalitalea cylindroides* DM1E8M, *Prevotella copri* D3E4B, and *Intestinibacter bartlettii* 80/4 isolates showed more notable inhibitory effects (average inhibition in the range of 55%–60%, *P*< .0001). Importantly, *B. adolescentis* L2-32 was identified as the strongest antagonist among all of the strains tested (63.6% average inhibition, Fig. 3; *P*< .0001). This was consistent with the 16S rRNA gene-based analysis described above, which had associated bifidobacteria with inhibition of *C. albicans* in the coculture experiments. Incubation with the bacterial growth medium alone (M2GSC) appeared to promote the growth of *C. albicans* slightly, although the effect was not statistically significant (Fig. 3), likely due to the presence of glucose in the medium, which *C. albicans* assimilates for growth.

Because of the strong inhibitory impact displayed by the *B. adolescentis* strain L2-32 supernatant, combined with the previously identified correlation of this species with strong antagonism against *C. albicans* in the coculture faecal incubation experiments described above, and the fact that this species is commonly detected in faeces from healthy adults (Matsuki et al. 2004), we next decided to focus on *Bifidobacterium* isolates and, in particular, on *B. adolescentis*, in more detail.

### Supernatants from specific *Bifidobacterium* strains inhibited *C. albicans* growth under anaerobic conditions

**Figure 4.**
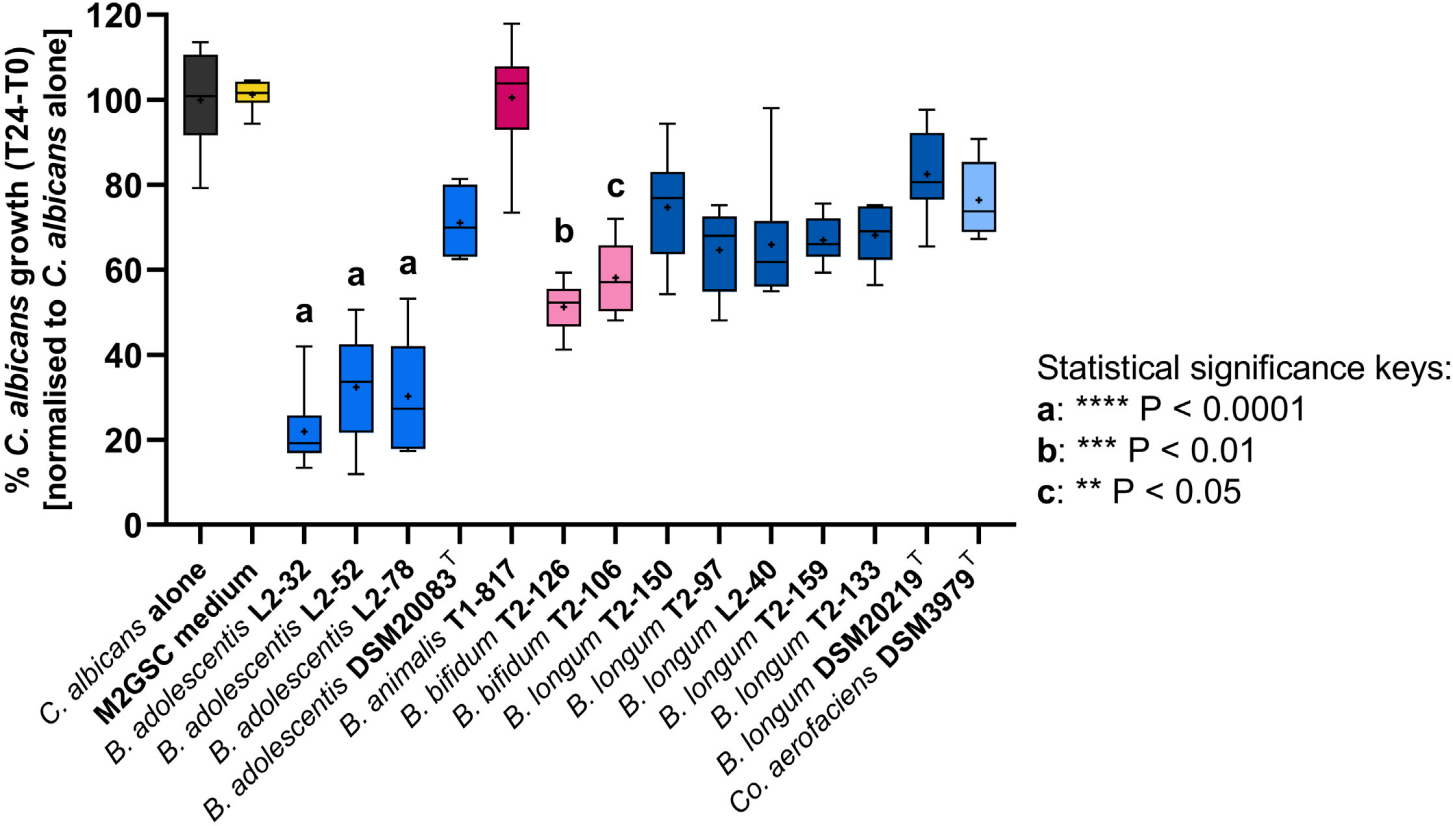
Impact of bifidobacterial and *Co. aerofaciens* culture supernatants on *C. albicans* growth under anaerobic conditions. The whisker boxplot represents % *C. albicans* growth (T24–T0) after incubation with culture supernatants from *Bifidobacterium* spp. or *Co. aerofaciens* strains isolated from healthy human donors. The crosses and central horizontal lines represent the mean and median, respectively, of six technical replicates per strain or for the *C. albicans*-only control (black). Strains are colour-coded by species. The Kruskal–Wallis test revealed a highly significant difference between samples (*P*< .0001), and Dunn’s *post hoc* test identified specific bifidobacterial isolates that exerted a significant inhibitory effect on *C. albicans* growth compared to the control.

To investigate whether different species of bifidobacteria inhibited the growth of *C. albicans in vitro*, four different bifidobacterial species, including one *B. animalis*, four *B. adolescentis*, two *B. bifidum*, and six *B. longum* strains, all isolated from the faeces of healthy humans (Table S2, Supporting Information), were screened for inhibition of *C. albicans* growth using the anaerobic assay described above. As *Co. aerofaciens* was correlated with less inhibitory effects on *C. albicans* in the faecal coculture work, we also included supernatants from one strain of this species in these experiments for comparative purposes. The supernatants of all bifidobacteria species tested resulted in 20%–80% *C. albicans* growth inhibition (relative to *C. albicans*-only growth in fresh NGY medium), except for *B. animalis* T1-817, which had no inhibitory activity (Fig. 4). In agreement with the earlier experiments, supernatants from three out of four *B. adolescentis* strains (L2-32, L2-52, and L2-78) most strongly inhibited *C. albicans* growth (*P*< .001; 68%–78% fungal inhibition compared to the no supernatant controls; Fig. 4). In contrast, the type strain *B. adolescentis* DSM 20083^T^ did not show a strong inhibitory effect, further indicating that the inhibitory activities may be strain-specific. Supernatants from *B. bifidum* T2-126 and T2-106 cultures were also significantly antagonistic against *C. albicans* in the anaerobic assay (*P*< .01 and *P*< .001, with 42%–49% fungal growth inhibition compared to the control, respectively). Finally, all representatives of the *B. longum* species tested showed a consistent, nonsignificant, mild inhibitory effect of approximately 20%–30% (Fig. 4).

### The inhibitory activity of bifidobacterial supernatants on *C. albicans* growth correlated with FA production and acidic pH

**Figure 5.**
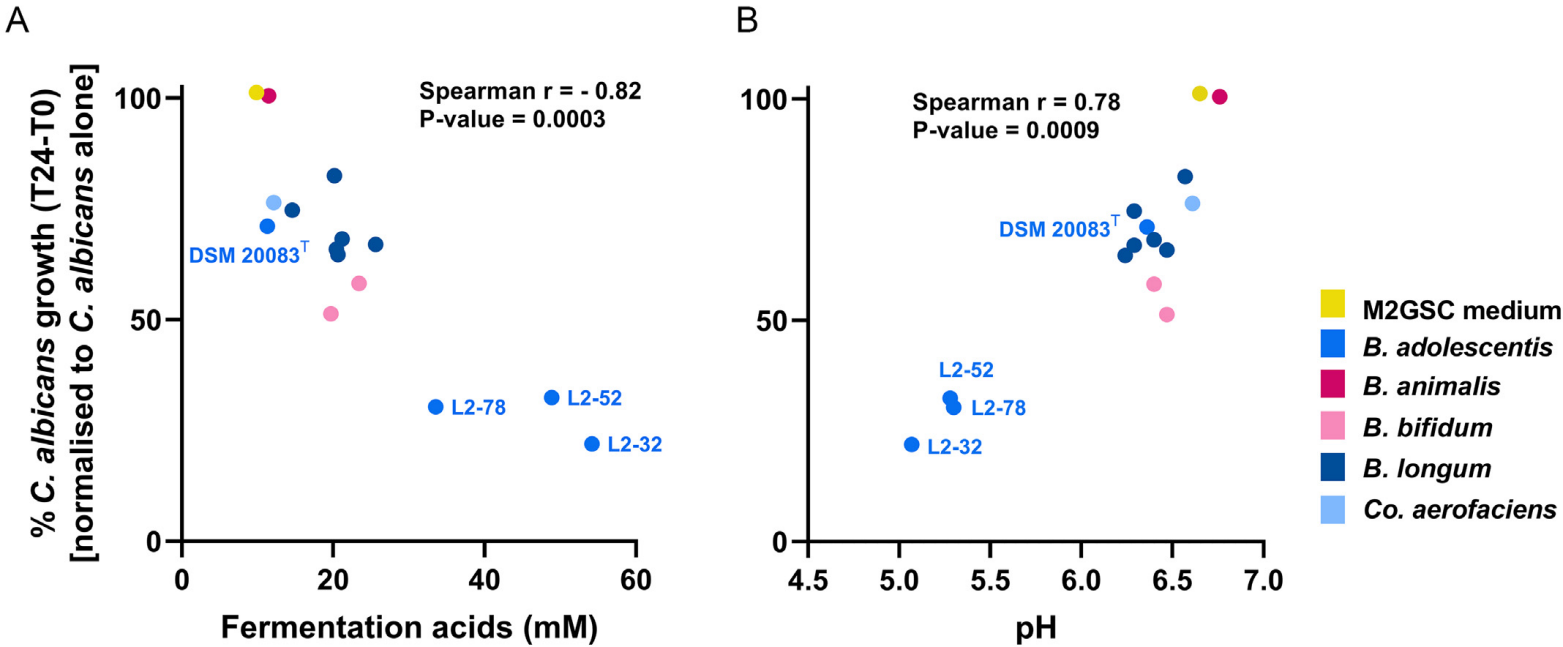
The inhibitory effect of *Bifidobacterium* and *Co. aerofaciens* isolates positively correlated with total concentration of FA and lower supernatant pH. Spearman correlation revealed that *C. albicans* inhibition was strongly associated with the FA concentration (A) and pH (B) of the bifidobacterial culture supernatants. Dots are colour-coded according to bacterial species, as per the key in the figure. *P*-values were corrected using the Benjamini, Krieger, and Yekutieli false discovery rate approach.

Having determined that culture supernatants from certain *Bifidobacterium* species from the human gut exert inhibitory activity against *C. albicans*, we next investigated the potential mechanisms underlying this phenomenon. As anticipated, quantification of the FA in the bifidobacterial supernatants used in the anaerobic assay revealed that the main organic acids produced by these strains were acetate, lactate, and formate (Table 1). *Bifidobacterium adolescentis* L2-32 produced the highest levels of the FA (38.1 mM acetate, 9.9 mM lactate, and 4.2 mM formate), followed by *B. adolescentis* L2-52 (20.7 mM acetate, 8.2 mM lactate, and 4.7 mM formate), and *B. adolescentis* L2-78 (31.2 mM acetate, 11.4 mM lactate, and 6.2 mM formate; Table 1). The bifidobacterial strains producing the highest total concentrations of these FA, therefore, also displayed the strongest antagonistic activity against *C. albicans* (Fig. 5). In contrast, we detected low concentrations of organic acids in noninhibitory strain supernatants, such as those from *B. animalis* T1-817 and from the *B. longum* strains (Table 1), suggesting that the inhibitory capacity of certain human gut bifidobacteria might be associated with the release of primary metabolites into the supernatant.

**Table 1.** Total and individual FA concentrations in the culture supernatants of bifidobacterial strains. In addition to FA profiles for each of the tested bifidobacterial, and *Co. aerofaciens*, strain supernatants, the corresponding supernatant pH, and % *C. albicans* growth (T24–T0; *n* = 6 technical replicates) are also shown. Table also shows Spearman correlation results, indicating that total FA, acetate, lactate and pH were all significantly associated with *C. albicans* inhibition.

Strain	% *C. albicans* growth	Acetate (mM)	Lactate (mM)	Formate (mM)	Total FA	pH
*B. adolescentis* L2-32	22.00	37.17	8.7	8.34	54.21	5.07
*B. adolescentis* L2-52	32.43	20.67	8.2	4.69	33.56	5.3
*B. adolescentis* L2-78	30.26	31.21	11.42	6.23	48.86	5.28
*B. adolescentis* DSM 20083^T^	71.13	6.05	3.1	2.16	11.3	6.36
*B. animalis* T1-817	100.48	7.64	0	3.81	11.45	6.76
*B. bifidum* T2-126	51.30	13.84	2.77	3.1	19.71	6.47
*B. bifidum* T2-106	58.17	16.22	4.07	3.12	23.41	6.4
*B. longum* T2-150	74.74	13.83	4.12	3.24	21.19	6.4
*B. longum* T2-97	64.68	17.62	5.45	2.53	25.6	6.29
*B. longum* L2-40	65.94	14.62	4.32	1.71	20.64	6.24
*B. longum* T2-159	67.03	14.02	4.59	1.55	20.17	6.57
*B. longum* T2-133	68.17	12.95	3.87	3.58	20.41	6.47
*B. longum* DSM 20219^T^	82.52	8.74	4.22	1.64	14.6	6.29
*Co. aerofaciens* DSM 3979^T^	76.43	0.46	6.52	5.19	12.16	6.61
M2GSC medium	101.21	9.87	0	0.72	10.59	6.65
Spearman coefficient		−0.87	−0.62	−0.46	−0.82	0.78
Corrected *P*-value (two-tailed)		.0001	.0043	.0175	.0002	.0003

To assess whether the inhibitory activity observed in the anaerobic assay was associated with the production of FA, we performed Spearman’s coefficient analysis by plotting the % growth of *C. albicans* vs. the total amount of FA in the gut bacteria supernatants. We observed a strong positive correlation between total FA levels and fungal growth suppression (r = –0.82; Fig. 5A). Similarly, we noted a strong negative correlation between pH and *C. albicans* growth, with the lower pH correlating with reduced fungal growth (r = 0.78; Fig. 5B). We also calculated Spearman’s correlation coefficients for the main individual FA produced by the *Bifidobacterium* strains (Table 1). This analysis revealed that acetate, lactate, and formate concentrations were all significantly associated with *C. albicans* inhibition.

### Sensitivity of *C. albicans* to individual and combined FA, and pH extremes, under anaerobic growth conditions

**Figure 6.**
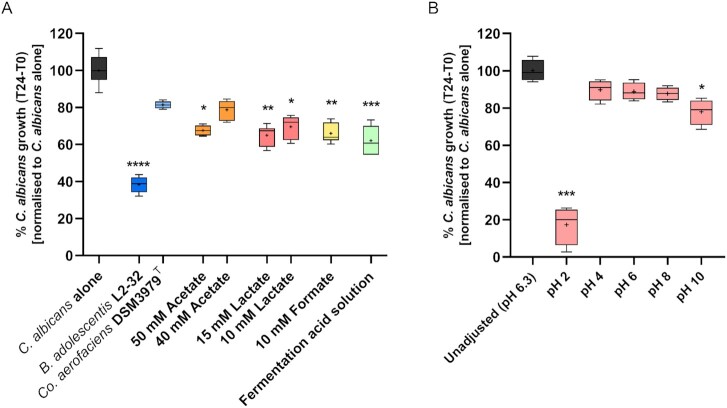
Impact of single and combined FA, as well as pH, on *C. albicans* growth under anaerobic conditions. (A) Individual FA and a mixed acid solution at concentrations detected in the most inhibitory (*B. adolescentis* L2-32) supernatant (40 mM acetate, 10 mM lactate, and 10 mM formate) were tested for their impact on the growth of *C. albicans*. The whisker boxplot includes the mean and median of six technical replicates as crosses and horizontal lines, respectively. The Kruskal–Wallis test indicated strong differences between the observed values (*P*< .0001); Dunn’s *post hoc* test revealed concentration-dependent inhibitory effects of the individual FA, with a particularly strong effect of 15 mM lactate and 10 mM formate, compared to the *C. albicans*-only control. (B) Effect of pH on *C. albicans* growth, under anaerobic conditions. pH values were adjusted by modifying NGY culture medium before filter-sterilization. The whisker boxplots show mean and median of four technical replicates. The Kruskal–Wallis test indicated significant differences between the observed values (*P* = .0024); Dunn’s *post hoc* testing indicated significant differences in fungal growth between the medium with unadjusted pH (pH 6.3, black), and pH 2 and pH 10. Significance values: ^****^P< .0001, *** *P*< .001, ** *P*< .01, and * *P*< .05.

We next tested the effect of individual and mixed FA solutions, at concentrations analogous to the previously observed highly inhibitory *B. adolescentis* supernatants (40–50 mM acetate, 10–15 mM lactate, and 10 mM formate), on *C. albicans* growth in the anaerobic assay. The FA mixture containing acetate, lactate, and formate significantly reduced *C. albicans* growth compared to the control over the incubation period (mean fungal inhibition of 38%, *P*< .001; Fig. 6A). Similarly, the individual FA showed a consistent suppressive effect on *C. albicans* growth (mean fungal inhibition of approximately 35% compared to controls), despite formate and lactate being added at lower concentrations than acetate (Fig. 6A). This may be related to the fact that lactate and formate are stronger acids (pKa around 3.8) than acetate (pKa of 4.8). However, of note, the extent of inhibition exerted by the individual and mixed FA solutions was inferior to the impact on fungal growth displayed by *B. adolescentis* L2-32 supernatants in the same test (Fig. 6A). This suggests the potential existence of additional inhibitory factors in the supernatant.

We then assessed the sensitivity of *C. albicans* to pH, by incubating the fungus in NGY culture medium adjusted to pH values ranging from 2 to 10. In contrast to the FA-based tests, pH values within the normal range of those detected in the lower GIT seemed to have little impact on *C. albicans* growth when tested as the sole variable (Fig. 6B). Indeed, fungal growth was only significantly decreased at extreme pH values, particularly at pH 2 (*P*< .001) and at pH 10 (*P*< .05), compared to the fungal growth in unadjusted NGY medium (Fig. 6B). This indicated that the suppression of *C. albicans* growth observed in the presence of culture supernatants is not driven solely by pH.

### Inhibition of *C. albicans* by bifidobacterial supernatants was mediated via the combined effects of pH and SCFAs

To further uncover the mechanisms underpinning the inhibitory capacity of the *B. adolescentis* strains tested, we next set up an anaerobic assay to study the effect of the following individual stressors on *C. albicans* growth: pH alone, exposure to a mixed solution of FA [45 mM acetate, 15 mM lactate, and 10 mM formate, to mimic the concentrations determined in the most inhibitory (*B. adolescentis* L2-32) supernatant], and bacterial culture supernatants. To better understand the combinatorial role of FA concentration and pH, we conducted the tests at different controlled pH values, in the range from 4 to 7, adjusting either the medium, or the test solution/supernatant.

**Figure 7.**
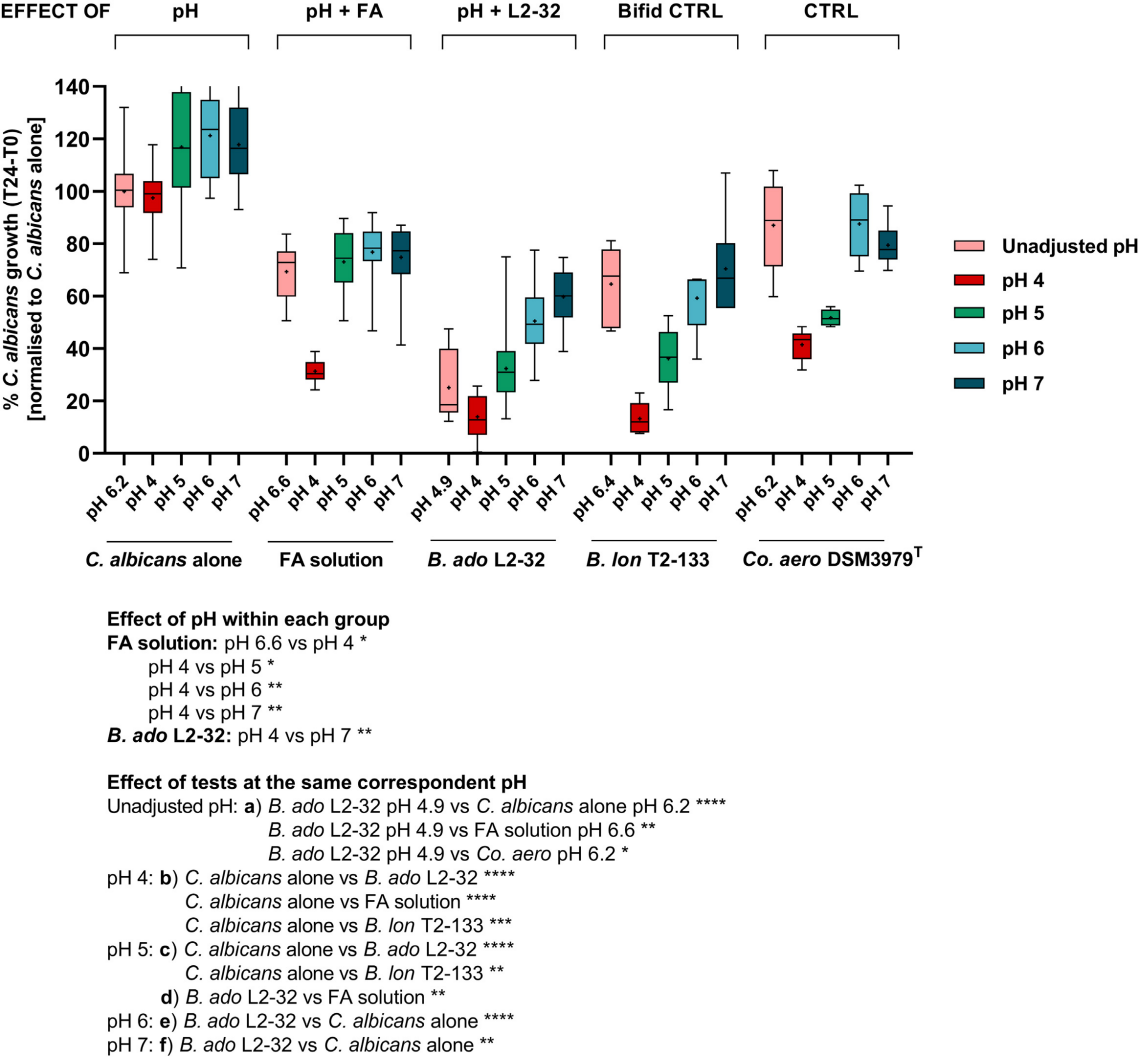
Cumulative impact of pH and FA on *C. albicans* growth under anaerobic conditions. The whisker boxplot shows *C. albicans* growth when tested at different controlled pH values, adjusting either the medium or the test solution/supernatant in the range from pH 4 to 7, under anaerobic conditions. Crosses and central horizontal lines represent the mean and median, respectively, of 12 technical replicates per test (*n* = 32 for *C. albicans* alone at pH 6.2, *n* = 18 for FA solution at all tested pH values, *n* = 24 for both *B. adolescentis* L2-32 and *Co. aerofaciens* DSM 3979^T^ at all tested pH values). The Kruskal–Wallis test indicated highly significant differences between groups (*P*< .0001); Dunn’s *post hoc* test, comparing the observations against each other, indicated significant differences within each group at different pH values (colour-coded as per legend) and between groups at the same corresponding pH, indicated separately in the Figure legend as ‘effect of pH’ and ‘effect of tests at the same correspondent pH’, respectively. Significance: ^****^P< .0001, *** *P*< .001, ** *P* < .01, and  * *P*< .05. *B. ado*, *Bifidobacterium adolescentis*; *B. lon*, *Bifidobacterium longum*; *Co. aero*, *Collinsella aerofaciens*.

Consistent with the previous observations (Fig. 6), *C. albicans* was highly resilient to the pH range tested under anaerobic conditions (Fig. 7). Critically though, altering the pH significantly impacted the inhibitory activity of the tested supernatants, and the FA mix. In all cases, these treatments were most inhibitory at the lowest pH tested (pH 4), and progressively lost potency against *C. albicans* as the pH increased (Fig. 7). This indicated that pH and FA combine to produce an inhibitory effect on *C. albicans*.

Of note, the antagonistic effect of the *B. adolescentis* L2-32 supernatant was significantly more pronounced than that of the SCFA solution at pH 5, as well as to that of a solution with an unadjusted pH value (Fig. 7), again suggesting that the bacterial supernatant might contain additional, but currently unidentified, inhibitory factors.

## Discussion

*Candida albicans* is a major clinical challenge because of high mortality in susceptible patients, emerging resistance against antifungal and sanitizing agents, and the limited availability of additional therapeutic options (Pfaller et al. 2000). Alternative strategies to reduce carriage and dissemination of *C. albicans* in the gut should, therefore, be explored. The healthy intestinal microbiota is an appealing source of novel treatments, considering the well-established role it plays in protecting against systemic candidiasis by hindering fungal expansion and pathogenic initiation in the gut (Kennedy and Volz 1985a,b).

It is noteworthy that the level of *C. albicans* inhibition observed in some of our faecal coculture experiments (Fig. 1) were much greater than those observed with single-species supernatants (Fig. 3), indicating that the cumulative activities of the gut microbiota may be more powerful inhibitors of *C. albicans* than individual constituent species. Nonetheless, we also showed that the degree of inhibition from mixed microbial communities is highly variable, strongly indicating that some gut microbiota species are likely to be inherently more inhibitory against *C. albicans* than others. However, the gut microbiota is extremely complex and it is currently largely unknown which components are most likely to be potent inhibitors of *C. albicans* in the gut. We demonstrate here that *B. adolescentis* culture supernatants exert strong inhibitory activity against *C. albicans* under anaerobic conditions *in vitro*, and identified an inhibitory effect of secreted bacterial FA and prevailing pH on *C. albicans* growth. These observations were in agreement with our DNA sequence-based analysis correlating the presence of *B. adolescentis* with the inhibition of *C. albicans* in mixed coculture with faecal microbiota samples, under conditions mimicking the human colonic environment.

The *Bifidobacterium* genus is dominant in the colon of breast-fed infants (Khonsari et al. 2016, Yatsunenko et al. 2012) and it also accounts for approximately 5% of the microbiota in adults, of which the species *B. adolescentis* is a prevalent representative (Reuter 1963). Importantly, *B. adolescentis* is also enriched following consumption of resistant starch (Ze et al. 2012), and produces high amounts of organic acids as a result of carbohydrate fermentation (Table 1). Despite the relatively low proportional abundance of this genus in the total microbiota in adults, it has potential health benefits for the host (Fukuda et al. 2011, Rivière et al. 2014, Rossi et al. 2005). Aside from FA production, bifidobacteria have also been demonstrated to induce the anti-inflammatory cascade (Lammers et al. 2003, Meng et al. 2016), and improve colonization resistance against common food-borne pathogens such as *E. coli* O157: H7 and *Salmonella enterica* serovar Typhimurium (Fukuda et al. 2011, Makras and De Vuyst 2006, Ventura et al. 2016). In addition, *B. adolescentis* colonizes the epithelial mucus layer and may therefore out-compete pathogens for adhesion sites on the gut epithelium (Tan et al. 2016, Ventura et al. 2016), potentially impacting the biofilm formation that can be an important virulence factor in *C. albicans* (Gulati and Nobile 2016).

Importantly, previous work in immunosuppressed gnotobiotic mice showed that supplementation with the bifidobacterial species *B. infantis* and *B. lactis* suppressed *C. albicans* growth in the intestines of the mice, and reduced systemic candidiasis (Wagner et al. 1998). Bifidobacteria were also recently predicted as major antagonists against *C. albicans* in an *in silico* model of intermicrobial interactions in the human gut (Mirhakkak et al. 2020). Bifidobacteria such as *B. adolescentis* may, therefore, be promising candidates for novel microbiota-based therapeutics aimed at enhancing colonization resistance. Several clinical trials have reported some efficacy of probiotic supplementation of *Bifidobacterium* and *Lactobacillus* spp. in reducing *C. albicans* intestinal colonization and preventing invasive fungal sepsis in infants following antibiotic treatment (Romeo et al. 2011, Roy et al. 2014). Furthermore, because *B. adolescentis* is a common member of the adult gut microbiota (present in up to 83% of healthy adults; Junick and Blaut 2012, Matsuki et al. 1999) and responds to changes in the diet, the growth and metabolic activities of this species could potentially be modulated *in vivo* by prebiotic supplementation.

Aside from bifidobacteria, other gut bacterial taxa are also likely worthy of further study. For example, we also observed inhibitory effects against *C. albicans* by a number of other gut bacterial species (Fig. 3). Wider screening of gut bacterial isolates is, therefore, highly likely to identify additional candidates with pronounced anti-*Candida* activity. In contrast, we also identified bacterial supernatants with little effect on *C. albicans* growth, such as those derived from *Flavonifractor plautii* and *Hungatella hathewayi*. This is consistent with reports that the relative abundances of these two bacterial species are correlated with *C. albicans* levels in faecal samples from cancer patients (Mirhakkak et al. 2020). Our results also highlight that different strains of the same gut bacterial species may have varying impacts on *C. albicans* growth (Fig. 3). Better understanding of the mechanistic basis for some of the putative interactions, both beneficial and detrimental, between specific gut bacteria and *C. albicans* may help to prioritize candidates for further study as potential novel therapeutics.

Although specific members of the gut microbiota appear to be particularly inhibitory against *C. albicans* it must be acknowledged, however, that the *in vitro*-based inhibitions observed here may not be mirrored *in vivo*, where the inhibitory gut anaerobes must simultaneously compete with the dense and complex wider gut microbiota. Interactions between members of the gut microbiota might enhance or restrict any secreted anti-*Candida* activities via mechanisms such as competitive exclusion of the putatively beneficial gut bacteria, or suppression of metabolic activity. Further development of any single-species therapeutics will, therefore, require extensive testing for efficacy *in vivo*. Indeed, work by Maldonado-Gómez et al. (2016), using *B. longum* as an exemplar probiotic, has shown quite clearly that the engraftment success of such probiotics depends in large part on niches being available for colonization, and that this is strongly dependent on the baseline microbiota at time of probiotic consumption. This may mean that efficacy of supplemented therapeutics will vary between individuals.

Furthermore, relatively little is currently known about the potential health impacts of the vast majority of gut anaerobes. There is also some evidence to suggest that consumption of single-species therapies can impact on wider microbiota dynamics after supplementation into the gut ecosystem, and might actually delay microbiota reconstitution following perturbation episodes (Suez et al. 2018). Therefore, in addition to efficacy testing, extensive safety testing will likely also be required, particularly if these gut bacteria were to be used in the immunocompromised individuals who are typically at greatest risk of severe/systemic *C. albicans* infection.

A key mechanistic result of the current study is demonstrating the combinatorial effect of FA and pH on the growth of *C. albicans*. Our findings are consistent with previous work indicating that the protonated form of weak acids freely permeate and accumulate inside microbial cells, causing dissipation of the proton motive force (Axe and Bailey 1995), triggering energetically expensive stress responses (Henriques et al. 1997), and perturbation of essential metabolic reactions (Cottier et al. 2015, Jacobsen et al. 2018, Lourenço et al. 2018). Gut microbiota FA are, therefore, thought to play important roles in limiting *C. albicans* intestinal colonization *in vivo* (Guinan et al. 2019, Huang et al. 2011), and a decrease in caecal SCFA concentrations following antibiotic treatment is associated with increased *C. albicans* load in the faeces in mouse models (Bohnhoff et al. 1964, Guinan et al*.*^2019^).

In agreement with our observations presented here, *C. albicans* was shown to be susceptible to formate (Mirhakkak et al. 2020) and acetate, at concentrations of over 30 mM, *in vitro*, and the effect is aggravated by microaerophilic conditions (Lourenço et al. 2018). Further, acetate inhibits hyphal morphogenesis of *C. albicans*, and this inhibition of hyphae formation could, therefore, reduce fungal translocation through the epithelial barrier (Guinan et al. 2019). In contrast, previous work has shown that lactate does not impair fungal growth at concentrations tested in our study, even at low pH values, and under aerophilic/microaerophilic conditions (Lourenço et al. 2018). Indeed, lactate is a potential energy source for *C. albicans* under hypoxic conditions, and is known to induce sustained fungal resistance to osmotic and cell wall stress, via cell wall remodelling (Ene et al. 2012a,b, 2015). Nonetheless, substantial lactate release (up to approximately 110 mM), among other factors, is postulated to contribute to lactic acid bacteria-mediated colonization resistance to *C. albicans* in the vaginal tract (Köhler et al. 2012, Zangl et al*.*^2020^).

Importantly, the total FA and acetate concentrations that *C. albicans* cells were exposed to in this study are physiologically relevant for regions of the human GIT such as the proximal colon (Cummings and Macfarlane 1991). Indeed, total SCFA levels may reach up to 200 mM in the proximal colon (Cummings and Macfarlane 1991), suggesting that inhibition of *C. albicans* growth mediated by total FA may be greater than indicated by our pure culture studies, and may represent a key mechanism of colonization resistance to this opportunistic fungus. In contrast, the concentrations of formate and lactate detected here in the bifidobacterial culture supernatants appear to be slightly higher than those detected in human faecal samples, where they do not usually exceed 5–10 mM, as they are absorbed by the host or utilized by other bacteria (Duncan et al. 2007, Hove et al. 1994). Additionally, the finding that supernatants were often more inhibitory than defined FA mixtures (Figs 6 and 7) suggests that additional, but currently unidentified, antifungal compounds may be produced by some gut anaerobes. This may be a worthwhile avenue for further study.

## Conclusions

In this *in vitro* study we showed that the degree of *C. albicans* growth inhibition by mixed human faecal microbiota communities can vary greatly between individual faecal donors. Specific components, such as *B. adolescentis*, were identified as being more antagonistic against *C. albicans* than other tested gut microbiota species. Inhibitory activity was predominantly driven by the release of FA, and the concomitant drop in ambient pH. The potential for altering the gut microbiota composition, e.g. by consumption of probiotics such as *B. adolescentis*, or increasing *in vivo* SCFA concentrations by consumption of dietary fibres such as resistant starch, are worthy of further study to determine whether these can bolster colonization resistance against *C. albicans* in the gut.

## Supplementary Material

fiac095_Supplemental_Files
